# Supporting Adolescent Mothers to Make Infant Feeding Decisions: A Qualitative Evidence Synthesis

**DOI:** 10.1111/mcn.70098

**Published:** 2025-08-29

**Authors:** Rachmawati Widyaningrum, Anna Gavine, Nicola M. Gray, Albert Farre

**Affiliations:** ^1^ School of Health Sciences University of Dundee Dundee UK; ^2^ Bachelor Program of Nutrition Universitas Ahmad Dahlan Yogyakarta Indonesia

**Keywords:** adolescent mothers, breastfeeding, infant feeding decision‐making, infant feeding support, qualitative evidence synthesis, qualitative research, systematic review

## Abstract

During the perinatal period, mothers make decisions on how to feed their infants. Adolescent mothers can have additional challenges in the decision‐making process (e.g., lack of autonomy, lack of support from professionals). We conducted a qualitative evidence synthesis to explore adolescent mothers' experiences in making infant feeding decisions, identify their support needs, and understand the role of healthcare professionals in supporting them through this process. Following a systematic search, 51 studies were included. Thematic synthesis was used and identified themes and sub‐themes. The four themes are: autonomy and the roles of others; changes in feeding decision making; mothers' self‐efficacy in breastfeeding; and experiences of formal support from healthcare professionals. We found that adolescent mothers still have unmet support needs, highlighting the necessity for tailored assistance, including non‐judgmental help, follow‐up care and easily understandable informational materials to facilitate appropriate infant feeding decision‐making.

## Summary

- Infant feeding decision‐making is a continuous and dynamic process in which decisions might change during the perinatal period.
- Adolescent mothers' self‐efficacy and autonomy may be compromised during the postnatal period, underlining the importance of support during this period.
- There is a gap between the support provided by healthcare professionals and the support needs of adolescent mothers, and thus a need to develop interventions that meet the distinctive needs of adolescent mothers.

## 1 Introduction

During the perinatal period, which spans pregnancy and the year after childbirth (WHO 2022), mothers make decisions on how they will feed their infant, a process referred to as infant feeding decision‐making (Radzyminski and Callister 2016; WHO & UNICEF 2018). Decision‐making, in general, is defined as the act of choosing a course of action from a range of possible options (Chick et al. 2012). In the infant feeding context, this process is influenced by several factors, including advice from healthcare professionals, family and friends, sociocultural factors, and the role of the media (Matriano et al. 2022). Feeding decisions include exclusive or partial breastfeeding, or the use of breastmilk substitutes. In some cultures it may also include solid food. The process is dynamic and complex, and decisions can have significant long‐term health implications for both mothers and infants (Robinson and Fall 2012; Tahir et al. 2021). For example, exclusive breastfeeding can fulfil a child's nutritional requirements, reduce the risk of infection, prevent stunting, and enhance cognitive development (Horta and Victoria 2013; Rachmayanti et al. 2022; Victora et al. 2016). In contrast, early introduction of pre‐lacteal and formula feeding, may increase the risk of infection and hospital admission (Nguyen et al. 2020). Thus, there are significant implications for immediate and future individual and public health outcomes and demands on healthcare provision.

The prevalence of adolescent pregnancies remains high worldwide (Gardner et al. 2023). Despite reports indicating declines in all countries—particularly in high income countries—the Sustainable Development Goals continue to prioritise further reducing adolescent birth rates due to the significant health risks posed to mother–infant dyads, as well as to promote the overall well‐being of this population (WHO 2024).

Adolescent mothers are defined as girls aged 10–19 who have given birth (Leftwich and Alves 2017). Evidence suggests that young mothers are at greater risk of suboptimal infant feeding compared to older mothers (Benova et al. 2020; Jama et al. 2018), with younger mothers less likely to breastfeed exclusively for the first to 6 months (Buckland et al. 2020). Young mothers may face additional challenges making infant‐feeding choices (e.g., high dependency on others and lack of autonomy, insufficient support from health professionals) while still pursuing their life interests and developmental needs (e.g., returning to school) (Hannon et al. 2000; Jama et al. 2018; Kanhadilok and McGrath 2015; Merino et al. 2013).

Health professionals are expected to guide and support infant feeding decisions (Haiek et al. 2021; Unicef UK 2018). To do this effectively, health professionals need to have empathetic and non‐judgmental discussions with mothers, respecting their values, considering their circumstances, and helping them to reach their goals (Munro et al. 2019; Sheehan et al. 2013). These discussions also serve as education on how to make important health‐related decisions (Wambach and Koehn 2004).

Several studies have explored the experiences and factors influencing adolescent mothers' infant feeding decisions, consistently finding that these decisions are influenced by family and peer influence, structural barriers, persistent knowledge gaps, and limited engagement with healthcare professionals (Jama et al. 2018; Wambach and Koehn 2004). However, most research and reviews have focused on enhancing breastfeeding outcomes through interventions such as breastfeeding support, incentives and breastfeeding education (Buckland et al. 2020; Pinho‐Pompeu et al. 2023), rather than how decisions are made, that is, the decision‐making process. This review is original in that it focuses on the process of decision making, how adolescent mothers experience support for making decisions throughout the perinatal period and identifies opportunities for intervention along the decision‐making pathway. The aim of this qualitative evidence synthesis (QES) was to identify and synthesise evidence on adolescent mothers' experiences in making infant feeding decisions, their support needs, and understand the role of healthcare professionals in supporting this process.

## 2 Methods

The protocol for this QES is registered on PROSPERO (CRD42023427973). QES is a general term related to the systematic review of qualitative study evidence, commonly used to synthesise findings and develop a better understanding of issues (Flemming et al. 2019). It is reported using the ENTREQ (Enhancing transparency in reporting the synthesis of qualitative research) recommendations (Tong et al. 2012).

### 2.1 Search Strategy

The search strategy was developed based on the research questions, which were formulated using the SPIDER tool (Cooke et al. 2012). S (Sample): Adolescent mothers and healthcare professionals; PI (Phenomenon of Interest): Infant feeding decision‐making; D (Design): Interview or focus group or ethnography or grounded theory or phenomenological research; E (Evaluation): Perceptions, experiences, and support; R (Research type): Qualitative studies and qualitative data from surveys or mixed methods research.

Five electronic databases were systematically searched: MEDLINE (via EBSCO), CINAHL (via EBSCO), ASSIA, Web of Science, and Maternity and Infant Care (MIDIRS) (via Ovid). The databases were selected based on their relevance to maternity, nursing, allied health, and the social sciences, which may cover the topic of infant feeding decision‐making. See Supporting Information: Appendix 1 for Medline search strategy. Searches were conducted in June 2023 and updated in May 2025. Searches were not limited by publication date or language. Additional searches were conducted in the World Health Organization (WHO) regional databases. Reference lists from all included studies and relevant systematic reviews were scanned for eligible studies.

### 2.2 Eligibility Criteria

Studies were included if they were published in English and reported qualitative data on experiences of adolescent mothers of babies aged 0–12 months in making infant feeding decisions, or the experiences of healthcare professionals in supporting the process. WHO defined adolescent mothers as girls aged 10–19 who have given birth (Leftwich and Alves 2017). In this review, infant feeding decision‐making refers to the process by which a mother decides how to feed her baby. Infant feeding decision‐making support is defined as the provision of comprehensive, evidence‐based information that is free from commercial interests, respects the mother's rights, and enables her to make and implement her decision (WHO & UNICEF 2018).

The baby's age was identified based on the details provided in the participants' characteristics and the information included in the quotations. Only articles containing quotes that clearly indicated the baby was aged 0–12 months were included. No restrictions were applied regarding the baby's birth weight and term. Mixed methods studies were included if they reported qualitative data that was presented separately from any quantitative data. Studies involving participants with health conditions or specific medical care needs for either mother and babies were excluded from this review. We excluded studies published in languages other than English because translation resources were not available.

### 2.3 Selection Process

Two reviewers (RW and AF/AG/NG) independently screened titles and abstracts, followed by full texts, against the eligibility criteria. If full‐text articles were unavailable, RW sought assistance from the University of Dundee librarian and attempted to contact the article's author for access. Any disagreements during the screening process were resolved through discussion with a third reviewer. The selection process was managed using Rayyan (Ouzzani et al. 2016).

### 2.4 Data Extraction and Quality Appraisal

A data extraction form was used to extract study aims, setting, participant details, methods and study findings related to the research questions. Study authors were contacted if there was any missing information.

Included articles were critically appraised by two reviewers (RW and AF) using the Critical Appraisal Skills Programme (CASP) qualitative checklist (CASP 2018). Any discrepancies were resolved by discussion with a third reviewer. Studies were not excluded based on the quality/adequacy of the reporting.

### 2.5 Data Synthesis

Thematic synthesis (Thomas and Harden 2008) was used to synthesise the qualitative research findings. First, the included study files were uploaded to the NVIVO 14 software (Lumivero 2023), which was used to manage the data during the synthesis process. The data synthesis process comprised three stages. Initially, the first author (RW) conducted data familiarisation before performing line‐by‐line coding of the text. The codes were generated from the findings of the included studies that were relevant to the research questions. In the second stage, following the initial inductive analysis that generated open codes, the first author developed the descriptive themes. A second and third reviewer (AF, AG and NG) critically revised these themes until a final version was agreed upon by the review team. In the final stage, analytic themes were identified by examining relationships between the descriptive themes, grouping them, and synthesising the findings (Thomas and Harden 2008).

## 3 Results

### 3.1 Description of the Studies

**Figure 1:**
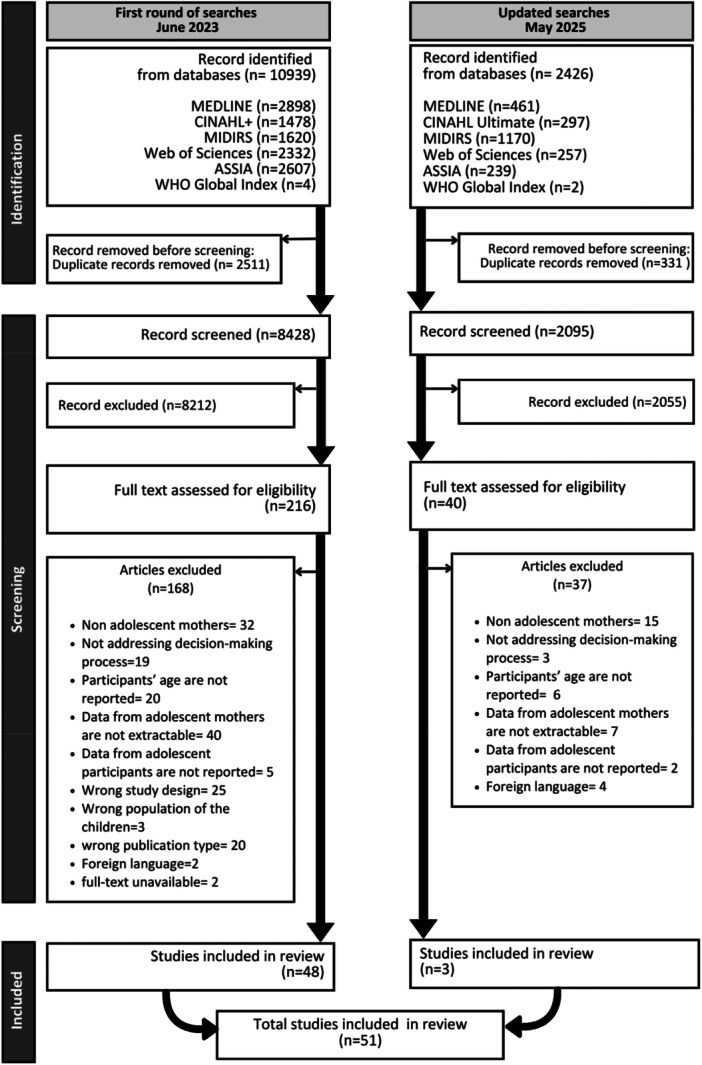
PRISMA flowchart for the systematic review.

After combining the search results from both searches, 2842 duplicate records were removed. A total of 10,523 records were assessed against the selection criteria. Of these, 10,267 articles were excluded at the title and abstract stage. A total of 256 full‐text articles were screened, and 205 articles were excluded. Reasons for exclusion included participants not being adolescent mothers, adolescent mothers' data not being extractable, and ineligible study designs. Details of the exclusion reasons are shown in Figure 1. The final number of studies included was 51.

### 3.2 Participants and Settings

Fifty‐one studies were included in the final synthesis, published between 1987 and 2025. Most studies were qualitative (*n* = 41); and 10 were mixed methods. Studies were conducted in the United States (*n* = 15), Canada (*n* = 2), Brazil (*n* = 6), Peru (*n* = 1), Columbia (*n* = 1), UK (*n* = 11), South Africa (*n* = 3), Ghana (*n* = 2), Sierra Leone (*n* = 1), Tanzania (*n* = 1), Australia (*n* = 1), New Zealand (*n* = 1), Turkey (*n* = 1), Indonesia (*n* = 2), India (*n* = 1), Thailand (*n* = 1) and Vietnam (*n* = 1). The adolescent mothers' age in this review ranged from 10 to 20 years. Of the 51 included studies, only six explicitly focused on infant feeding decision making. The remaining studies had a wider focus (e.g., breastfeeding, maternal health, infant feeding) but did include some information on infant feeding decision making. Five articles focused on low socioeconomic settings. The summary of the characteristics of included studies is presented in Supporting Information: Appendix 2.

### 3.3 Study Quality Assessment

In general, the research aim was well reported. However, there was insufficient description of methods and consideration of the participant‐researcher relationship was minimal. Table 1. provides a summary of our quality appraisal. Supporting Information: Appendix 3 provides further details of quality appraisal for each study.

**Table 1:** Summary of quality assessment of included studies (*n* = 48).

No.	Questions	Yes		Can't tell		No	
		*n*	%	*n*	%	*n*	%
1.	Was there a clear statement of the aims of the research	50	98.04	0	0.00	1	1.96
2.	Is a qualitative methodology appropriate	51	100.00	0	0.00	0	0.00
3.	Was the research design appropriate to address the aims of the research?	26	50.98	25	49.02	0	0.00
4.	Was the recruitment strategy appropriate to the aim of the research?	27	52.94	23	45.10	1	1.96
5.	Was the data collected in a way that addressed the research issue?	34	66.67	17	33.33	0	0.00
6.	Has the relationship between researcher and participants been adequately considered?	9	17.65	5	9.80	37	72.55
7.	Have ethical issues been taken into consideration?	37	72.55	10	19.61	4	7.84
8.	Was the data analysis sufficiently rigorous?	30	58.82	19	37.25	2	3.92
9.	Is there a clear statement of findings?	37	72.55	11	21.57	3	5.88

### 3.4 Findings

**Figure 2:**
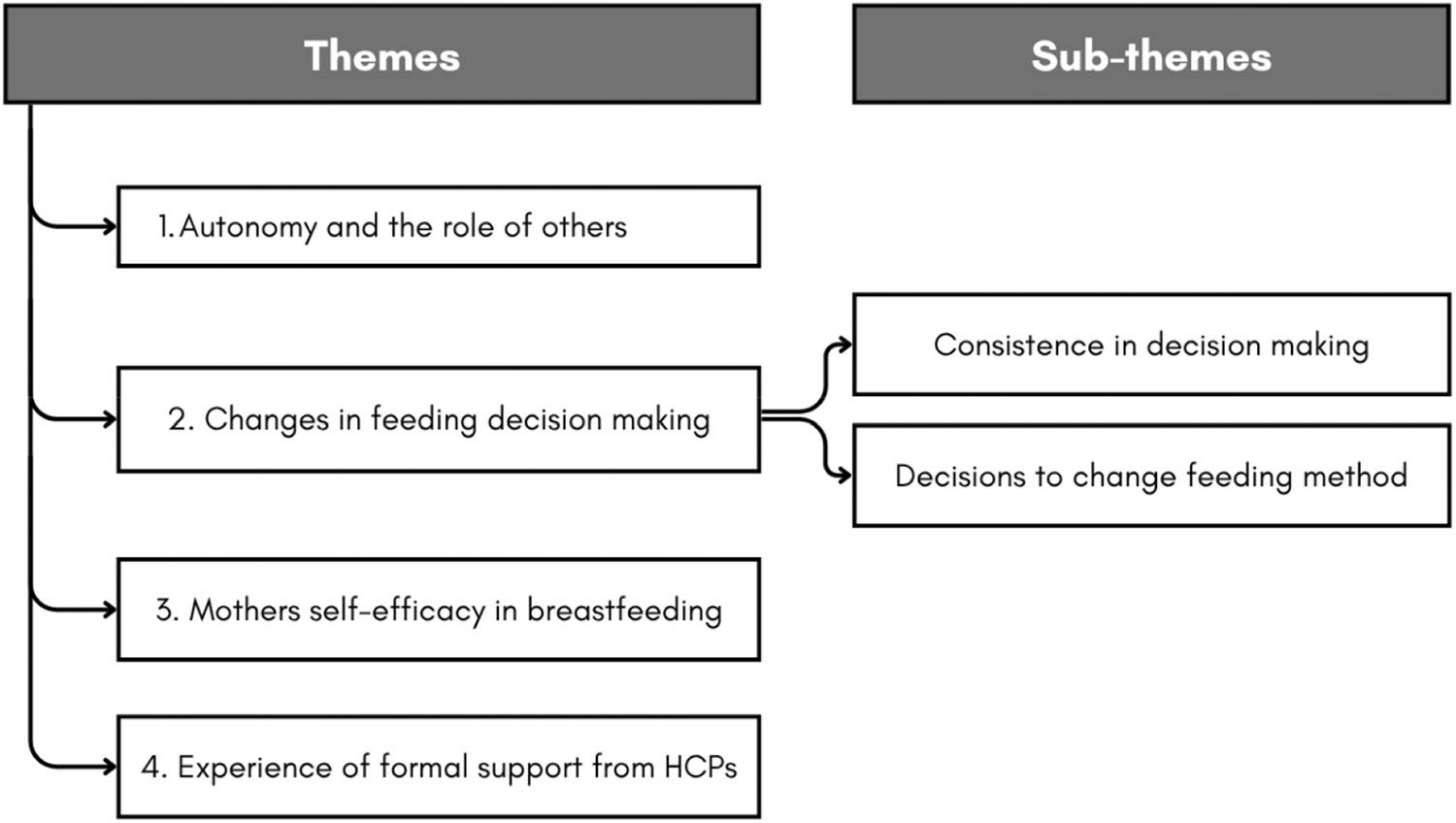
The list of themes and sub‐themes.

The analysis resulted in four overarching themes and subthemes: (1) Autonomy and the roles of others; (2) Changes in feeding decision making; (3) Mothers' self‐efficacy in breastfeeding; and (4) Experiences of formal support from healthcare professionals. The list of themes and sub‐themes are presented in Figure 2. The distribution of included studies underpinning each theme is shown in Table 2.

**Table 2:** The distribution of included studies underpinning each theme.

No.	Author	Theme 1	Theme 2	Theme 3	Theme 4
1.	Acheampong et al. (2020)	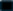	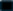		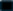
2.	Amekpor et al. (2025)		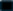		
3.	Arthur et al. (2007)		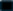	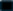	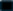
4.	Astuti et al. (2021)	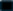	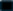	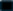	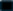
5.	Benson (1996)			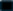	
6.	Bentley et al. (1999)	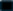	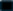		
7.	Bernie (2014)				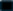
8.	Bettison (2014)				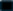
9.	Breevort et al. (2021)	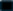			
10.	Concha and Jovchelovitch (2021)	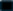			
11.	Condon et al. (2013)	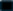	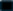	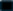	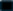
12.	Cooper et al. (2019)	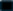			
13.	Debnath et al. (2021)	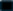	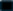		
14.	Duong et al. (2005)			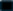	
15.	Dykes et al. (2003)	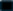	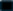	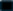	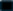
16.	Dyson et al. (2010)	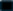	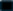		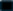
17.	Erfina et al. (2019)		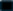		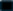
18.	Hannon et al. (2000)	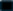	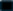	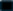	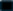
19.	Harner and McCarter‐Spaulding (2004)	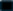			
20.	Hunter (2012)				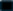
21.	Hunter and Magill‐Cuerden (2014)	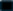	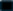	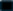	
22.	Hunter et al. (2015)				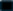
23.	Hunter‐Adams et al. (2022)				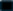
24.	Jama et al. (2017)			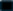	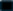
25.	Jama et al. (2018)	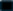		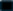	
26.	Kocturk (1987)			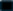	
27.	Leeming et al. (2015)				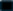
28.	Locklin (1995)				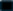
29.	Mazza et al. (2015)	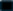	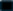		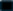
30.	Merino et al. (2013)		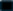		
31.	Monteiro et al. (2014)		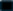		
32.	Moran et al. (2006)				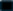
33.	Morrison et al. (2008)	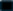			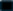
34.	Nelson (2009)	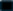			
35.	Nelson and Sethi (2005)	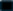			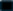
36.	Nesbitt et al. (2012)	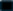		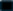	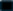
37.	Nuampa et al. (2018)	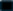		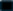	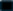
38.	Oliveira et al. (2016)	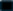	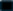	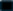	
39.	Pentecost and Grassley (2014)				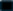
40.	Raisler (2000)	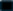			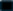
41.	Rothstein et al. (2020)				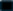
42.	Severinsen et al. (2024)	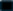			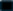
43.	Smith et al. (2012)	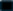	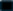	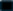	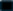
44.	Spear (2006)			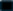	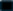
45.	Spindola et al. (2014)	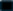			
46.	Tomeleri and Marcon (2009)	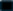		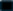	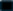
47.	Tucker et al. (2011)			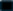	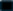
48.	Wambach and Cohen (2009)			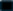	
49.	Wambach and Koehn (2004)	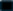			
50.	Wambach et al. (2016)			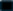	
51.	Yas et al. (2024)	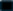			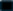

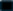
 = Covered.

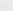
 = Not covered.

#### 3.4.1 Autonomy and the Role of Others

There were differences between the adolescent mothers perceived autonomy and the roles of others in their infant feeding decisions during the pre and postnatal period. In the prenatal period, some pregnant mothers felt that the decision was theirs, indicating high levels of perceived autonomy (Dyson et al. 2010; Hannon et al. 2000; Nelson 2009; Raisler 2000; Wambach and Koehn 2004), for example: “My mom said it would hurt too much, but it's my body and I wanted to breastfeed.” (Hannon et al. 2000, 404).

The included papers provided very few findings about the detailed decision‐making process. Most studies only mentioned that adolescent mothers relied on vicarious experiences from close family members or friends (Acheampong et al. 2020; Condon et al. 2013; Morrison et al. 2008; Smith et al. 2012), as well as information about the risks and benefits of each infant feeding method (Acheampong et al. 2020; Condon et al. 2013; Hannon et al. 2000; Hunter and Magill‐Cuerden 2014; Monteiro et al. 2014; Nuampa et al. 2018; Smith et al. 2012), as the basis for their decision‐making.

However, after giving birth, adolescent mothers reported less autonomy, with more involvement from others in the decision‐making process (Acheampong et al. 2020; Astuti et al. 2021; Benson 1996; Bentley et al. 1999; Bernie 2014; Concha and Jovchelovitch 2021; Condon et al. 2013; Cooper et al. 2019; Debnath et al. 2021; Dykes et al. 2003; Hannon et al. 2000; Harner and McCarter‐Spaulding 2004; Hunter‐Adams et al. 2022; Hunter et al. 2015; Jama et al. 2017, 2018; Kocturk 1987; Mazza et al. 2015; Merino et al. 2013; Morrison et al. 2008; Nesbitt et al. 2012; Nuampa et al. 2018; Oliveira et al. 2016; Raisler 2000; Rothstein et al. 2019; Severinsen et al. 2024; Smith et al. 2012; Tomeleri and Marcon 2009; Yas et al. 2024). Many adolescent mothers felt they had no autonomy to make feeding decisions after giving birth:

…I was like had no autonomy to take care of my own baby (pause), especially when against my mother‐in‐law (pause). I was placed as a person who did not know nothing about breastfeeding and infant feeding (pause). I was heartbroken (pause) when I found my 7 days old baby had been fed with a mashed banana by mother‐in‐law (pause).

(Astuti et al. 2021, 42)

The most influential persons for adolescent mothers were their close family members (Acheampong et al. 2020; Astuti et al. 2021; Bentley et al. 1999; Concha and Jovchelovitch 2021; Dykes et al. 2003; Hannon et al. 2000; Harner and McCarter‐Spaulding 2004; Jama et al. 2018; Mazza et al. 2015; Merino et al. 2013; Morrison et al. 2008; Nesbitt et al. 2012; Nuampa et al. 2018; Oliveira et al. 2016; Tomeleri and Marcon 2009). Most adolescent mothers reported following the baby's grandmother's advice on infant feeding: “My mother told me to do so” (Bentley et al. 1999, 1094), even when the advice was against the health recommendation, for example giving additional solid foods: “She (the child's grandmother) gave porridge too […] so as soon as they were born (children's great‐grandmother), she gives them porridge (Oliveira et al. 2016, 1258).”

Some healthcare professionals took a paternalistic role, with mothers reported that the healthcare professionals made the decision on their behalf (Benson 1996; Dykes et al. 2003; Hunter et al. 2015; Mazza et al. 2015; Raisler 2000; Rothstein et al. 2019; Smith et al. 2012), putting the adolescent mother in a passive role:

When [the nurse] came to my house to do the little check‐up she weighed him. And she was like, “Let's just try to see if he gets any bigger just doing one bottle.” And she fed him a bottle, and he gained two ounces just like that. So, I didn't ask nobody. I thought it was OK. But she was like, “It's just best to put him on the bottle.

(Smith et al. 2012, 8)

Some professionals went further and went against mothers' wishes by providing formula milk:

I said no bottles, and they would like force bottles on me, I think because I was younger too…. They gave her a bottle without even waking me up to ask me, because they said I was too weak after a hard labour. They could have come in and asked…I mean, I had made it specifically clear! I had told every single person that walked in there…I want this baby completely breastfed. That upset me more, I think, than the entire hospital stay.

(Raisler 2000, 256)

#### 3.4.2 Changes in Feeding Decision Making

##### 3.4.2.1 Consistence in Decision Making

Throughout the perinatal period, some adolescent mothers maintained their original feeding decision (Amekpor et al. 2025; Benson 1996; Concha and Jovchelovitch 2021; Cooper et al. 2019; Hannon et al. 2000; Jama et al. 2018; Mazza et al. 2015; Merino et al. 2013; Monteiro et al. 2014; Nelson 2009; Nelson and Sethi 2005; Nesbitt et al. 2012; Nuampa et al. 2018; Smith et al. 2012; Spindola et al. 2014; Tomeleri and Marcon 2009; Tucker et al. 2011; Wambach et al. 2016; Wambach and Cohen 2009), for some this was to exclusively breastfeed:

I gave, since the beginning, a little cracked right breast [breast], but even so I was, to get hurt, but then relieved, was up hottie, today is really hot now no longer feel pain, and I love breastfeeding.

(Spindola et al. 2014, 419)

and for others it was to formula feed:

I don't like to take out my breasts in public and feed the baby, I don't like it. I can't, but I do see some other people doing it but no I cannot imagine myself doing it, so I give him formula.

(Jama et al. 2018, 8)

##### 3.4.2.2 Decisions to Change Feeding Method

Most adolescent mothers reported changing their minds during the perinatal period, usually shifting from an initial decision to breastfeed to mixed/formula feeding (Arthur et al. 2007; Astuti et al. 2021; Benson 1996; Condon et al. 2013; Debnath et al. 2021; Dykes et al. 2003; Erfina et al. 2019; Hannon et al. 2000; Hunter and Magill‐Cuerden 2014; Jama et al. 2018, 2017; Leeming et al. 2015; Merino et al. 2013; Monteiro et al. 2014; Nesbitt et al. 2012; Nuampa et al. 2018; Oliveira et al. 2016; Smith et al. 2012; Tomeleri and Marcon 2009; Tucker et al. 2011; Wambach et al. 2016; Wambach and Cohen 2009), for example: “It was as if that wasn't enough. Then, I got NAN milk […] and gave it to him in a bottle […] he slept […] and soon I got my milk again […]” (Tomeleri and Marcon 2009, 274), or to add solids/pre‐lacteal: “Once I gave the baby solids he wasn't that interested [breastfeeding]” (Spear 2006, 111).

Fewer participants, shifted from formula feeding to breastfeeding (Condon et al. 2013; Hannon et al. 2000; Hunter and Magill‐Cuerden 2014; Locklin 1995; Nelson 2009; Tucker et al. 2011), for example:

I was not going to breastfeed her because I didn't want to. People don't just tell you do or don't breastfeed your baby! I didn't want to be in public, breastfeeding in public or switching rooms when company came over because she had to eat. You don't want to be pulling your clothes and stuff all around among people you don't know—even if you were covered. My mother had bottle‐fed us and had not encouraged me to breastfeed. But in the hospital, they made me try and then she got hooked to it and stopped taking her other milk and I had to breastfeed her. My doctor said, ‘Come on, let's breastfeed.’ He wouldn't allow me to give her any bottles and gave me a breastfeeding counsellor who told me why I should do it.

(Hannon et al. 2000, 405).

Decision changes such as this, again highlight the lack of autonomy experienced by some mothers.

#### 3.4.3 Mothers' Self‐Efficacy in Breastfeeding

There was a difference in mother's self‐efficacy pre and postnatally. This was particularly relevant for those breastfeeding, with mothers who demonstrated less self‐efficacy being more likely change their decision to breastfeed (Arthur et al. 2007; Astuti et al. 2021; Benson 1996; Condon et al. 2013; Dykes et al. 2003; Hannon et al. 2000; Hunter and Magill‐Cuerden 2014; Jama et al. 2017, 2018; Kocturk 1987; Nuampa et al. 2018; Oliveira et al. 2016; Smith et al. 2012; Spear 2006; Tomeleri and Marcon 2009; Tucker et al. 2011; Wambach et al. 2016; Wambach and Cohen 2009). In the prenatal period, some mothers reported that they believed they would be able to breastfeed:

When I first got pregnant, I said ‘I want to breastfeed, I hope I can breastfeed.’ I said, ‘even if it's hard, I still want to try and fight so I at least feed him for the first few months.

(Nesbitt et al. 2012, 6).

However, in the post‐natal period, many mothers felt low self‐efficacy due to breastfeeding challenges, such as issues with latch, pain, and tiredness (Arthur et al. 2007; Benson 1996; Dykes et al. 2003; Hannon et al. 2000; Hunter and Magill‐Cuerden 2014; Nuampa et al. 2018; Oliveira et al. 2016; Smith et al. 2012) for example: “I think the worst bit is when you start feeding and they're really sore….I tried creams and stuff and I was like “it's not working, it's not working” (Dykes et al. 2003, 394).

Adolescent mothers also reported milk insufficiency, breast refusal, or persistent crying (Astuti et al. 2021; Condon et al. 2013; Duong et al. 2005; Jama et al. 2017; Smith et al. 2012; Spear 2006; Tomeleri and Marcon 2009; Wambach et al. 2016; Wambach and Cohen 2009), for example:

I wanted to fully breastfeed my baby but I could not, even I tried to eat as much as I could. Breast milk was not enough for the baby and she cried for a whole day. I gave up after five days and started giving her some rice porridge.

(Duong et al. 2005, 340)

They also reported difficulties in maintaining breastmilk expression at school or workplace:

I quit once I went back to school because there was like no way I could do it. That would have been really hard…I mean, because I would have to like go pump like every two to three hour, and I just couldn't do that.

(Tucker et al. 2011, 7)

In contrast, mothers, who reported postnatal breastfeeding self‐efficacy (Benson 1996; Condon et al. 2013; Cooper et al. 2019; Mazza et al. 2015; Merino et al. 2013; Monteiro et al. 2014; Nelson and Sethi 2005; Spindola et al. 2014), were aware of the possibility of early difficulties and felt that the process was improving as they got used to it or they were able to get help to overcome the challenges:

I didn't want to breastfeed, my partner wanted me to, so I tried it and my midwife said, the first few days are going to be the worst but after the three days then it would be fine and ever since I've just breastfed. It's fine, it's brilliant.

(Condon et al. 2013, 159)

They also described receiving a positive response from the baby: “With him, he's obviously right there, and I can see every single detail of him and how he's doing and… he's satisfied. And it just makes me feel so good (Wambach et al. 2016, 106). This suggests that when self‐efficacy was high, mothers felt confident in their decision to breastfeed.

##### 3.4.3.1 Experience of Formal Support From Healthcare Professionals

The mothers in most of the included articles reported that they received infant feeding education from healthcare professionals in the prenatal period, postnatal period, or both, and most encouraged them to breastfeed (Astuti et al. 2021; Bettison 2014; Condon et al. 2013; Hunter‐Adams et al. 2022; Jama et al. 2017; Nesbitt et al. 2012; Pentecost and Grassley 2014; Raisler 2000; Smith et al. 2012), for example:

… She (midwife) was really helpful (pause); she taught me about how to breastfeed (pause); I remember that the midwife taught me starting from when I still got pregnant, and again she taught me just hours after my baby born.

(Astuti et al. 2021, 40)

However, the benefits of this support were overshadowed by their perceived unmet support needs, such as inconsistent advice: “They would say different things… all of them… which was confusing” (Dykes et al. 2003, 397), and rushed care provision that led to lack of opportunities for discussion:

She came in and she said ‘what do you want? We're busy.’ Sort of, like that! And I was thinking ‘alright, don't bother then! I'll try and do it [latch baby on] myself.’ And I just said ‘don't worry about it.’

(Hunter et al. 2015, 53)

Some mothers conveyed that the type of support they needed to feel comfortable was more personal:

Being personal, really really helps. Because, if like they're going to be saying like all kinds of medical terms and stuff then it's really hard to listen and then you kind of think it's coming right out of a book, and they don't know what it's like”

(Nelson and Sethi 2005, 618)

They also reported the need of easy‐to‐understand and more practical support:

‘As for me, I am young and have never given birth before but they (nurses and midwives) spend a lot of time telling us about the benefits of breastfeeding. They do not consider the fact that we never breastfed before. They must demonstrate to us how to actually breastfeed our babies and take time to repeat it once we delivered. This will definitely motivate us to exclusively breastfeed our babies for 6 months without problems.

(Acheampong et al. 2020, 3)

This suggests mothers do value practical support to breastfeed. However, across the studies, there was a lack of consideration of how the healthcare professionals could support adolescent mothers to make decisions as to how to feed their babies.

## 4 Discussion

This review focused on how adolescent mothers make infant feeding decisions, their experiences and support needs, and the role of healthcare professionals in supporting them. The findings of this review provide a deeper understanding of gaps in support during the decision‐making process, which might otherwise be overlooked when focusing solely on the outcomes of these decisions. Our findings highlight that decisions around infant feeding are influenced by a range of factors: social (family and friends), cultural, mothers' levels of autonomy, and structural (organisations/schools, education and healthcare support). When a decision to not breastfeed was made, very few mothers changed their mind towards breastfeeding, instead, most mothers reported a change from breastfeeding to other feeding methods (Kullmann 2021). This is consistent with findings from mothers over the age of 20 years (Rozensztrauch et al. 2022).

During the antenatal period, some mothers in this review showed confidence at being able to breastfeed (Conde et al. 2017). Previous studies have found that mothers with high self‐efficacy are more adept at overcoming challenges associated with breastfeeding, thereby enhancing the likelihood of sustained breastfeeding practices (Wu et al. 2022). However, mothers' self‐efficacy levels may change during the perinatal period, influenced by the differences between mothers' breastfeeding expectations and reality. Previous research has found that mothers who initially expected breastfeeding to be easy were not prepared for the challenges (Gálvez‐Adalia et al. 2022). These challenges can erode mothers' confidence, leading to an early introduction of formula feeding or breastfeeding cessation (Brown et al. 2011; Vilar‐Compte et al. 2022).

Our review found the role of others increased significantly during the postnatal period, along with the decrease of mother's autonomy. Hirani and Olson (2016) defined maternal autonomy in the breastfeeding context as the ability of a mother to independently make decisions; governed by her autonomy, agency, independence, and moral judgement; and influenced by factors such as her competency in motherhood, the support she has access to, the environment, and the options available to her. Involving others in the decision‐making process, such as close family and peers, might affect mothers' infant‐feeding intentions (Bootsri and Taneepanichskul 2017; Priscilla et al. 2021). In this regard, ongoing support is needed through mothers feeding journey at every level of decision‐making, including promoting autonomous decision making (Duran et al. 2022; Rahadian et al. 2022).

The baby's grandmothers were reported to affect the decision‐making process as well as influence adolescent mothers' ultimate decisions. A mother is potentially up to 12% more likely to initiate breastfeeding if her own mother has a positive attitude toward it and, conversely, a negative opinion can reduce breastfeeding likelihood by 70% (Negin et al. 2016), which may lead to formula or pre‐lacteal feeding (Chea and Asefa 2018). Additionally, living with or having frequent contact with grandmothers can negatively affect breastfeeding rates (Bica and Giugliani 2014; Emmott and Mace 2015). Given the potential influence of grandmothers, their inclusion in breastfeeding education and support may be warranted (Bica and Giugliani 2014). Breastfeeding promotions that involve grandmothers have shown beneficial effects on exclusive breastfeeding (Cresswell et al. 2019; Kimani‐Murage et al. 2021).

Adolescent mothers raised concerns about their unmet support needs. Irrespective of their feeding choices, adolescent mothers continue to seek support that enables them to enquire, discuss, and make decisions comfortably, while being satisfied with the choices they have made. Health literacy levels are generally lower in younger age groups, which may be attributed to reduced access to health information, developmental stage, and limited experience with the healthcare system, all of which can increase the risk of suboptimal infant feeding decisions (França et al. 2020). Lower health literacy underlines the need for improved support by providing continuous, user‐friendly, and understandable health interventions that account for adolescent learning pedagogies (Hall Moran et al. 2007).

Across the included studies there seemed to be a lack of support by healthcare professionals in the actual decision‐making process. In some cases, healthcare professionals went as far as to ignore the mothers' wishes and either gave the baby formula milk or refused to do so. Improved support also needs to address the perceived ‘judgmental nature’ of some health professionals, and their varying skills in providing adolescent‐friendly care (Peterson et al. 2012). Several ethical issues in service provision were identified in this review, including the provision of advice or information by healthcare professionals which were contrary to accepted health recommendations. Results of a previous study showed that at least one‐fifth of mothers received advice from healthcare professionals to feed their baby with breastmilk substitutes, mostly formula milk (Lutter et al. 2022). Therefore, better training and education regarding breastfeeding are essential to improve healthcare professionals' knowledge, attitudes, and skills, enabling them to provide the support mothers need (Gavine et al. 2017; Yang et al. 2018).

A key finding of our review is that, for some mothers, their autonomy was undermined by healthcare professionals who did not consult them about their infant feeding preferences or provided breast milk substitutes without their approval. These practices contradict UNICEF recommendations, which emphasise the importance of discussing infant feeding with mothers, providing evidence‐based information, and avoiding judgmental conversations (Unicef UK 2018). This highlights the need for safe, evidence‐based, person‐centred support for adolescent mothers during the infant feeding decision‐making process, both pre‐ and post‐birth.

### 4.1 Limitations

This review had limitations related to geographical context and participant parity. Most of the included studies were conducted in high‐income countries. Although potential differences between high‐income countries (HICs) and low‐ and middle‐income countries (LMICs) were acknowledged—highlighting limitations in the transferability of the findings—no clear patterns emerged to justify a separate analysis. Further research is needed in diverse settings to better understand adolescent mothers' experiences and support needs in the infant feeding decision‐making process.

As there are no previously published qualitative reviews on this subject, we aimed to undertake a comprehensive review without any restrictions on publication date. Although the settings and contexts of the included studies and participants have likely changed over time, we found no significant differences in the findings reported across the articles to justify limiting the review by date. Furthermore, given that the rate of change across countries is likely to vary, we sought to ensure inclusivity by capturing a broad range of contexts and time periods.

### 4.2 Implication for Practice

This review concludes that adolescent mothers have unmet support needs during the infant feeding decision making process. These needs may differ from those of adult mothers, particularly in relation to what support is required, when and how this is delivered. This highlights the need for tailored support, including non‐judgmental assistance, respectful care, and easily understandable informational materials to facilitate optimal infant feeding decision‐making.

Furthermore, the lack of autonomy among adolescent mothers, along with the influence from significant others such as the baby's grandmother, and unmet support needs from healthcare professionals underscores the importance of addressing these dynamics and relationships in developing effective support systems. Targeted interventions that recognise adolescent mothers' distinct support needs and their vulnerability and high dependency on others—such as involving the baby's grandmother and advocating for and respecting their own decisions—could improve infant feeding decision‐making and lead to better long‐term health outcomes for both mothers and babies. Healthcare professionals training is essential to enhance their skills to meet support needs of adolescent mothers.

### 4.3 Implication for Future Research

Future research could address the gaps identified in this review, including how the decision‐making process evolves during the perinatal period. Future studies could also explore a broader range of infant feeding decisions; not only breastfeeding and formula feeding, but also pre‐lacteal feeding, early introduction of solids, and donor milk. Longitudinal research might be useful to better understand the dynamic nature of the decision‐making process. Additionally, research focused on developing formal support systems that are tailored to the specific needs of adolescent mothers and assist them in the decision‐making process is essential.

## Author Contributions

Rachmawati Widyaningrum, Anna Gavine, and Albert Farre developed the review protocol development and undertook article screening. Rachmawati Widyaningrum, Albert Farre, and Nicola M. Gray analysed the data and developed the themes and results. Rachmawati Widyaningrum, Anna Gavine, Nicola M. Gray, and Albert Farre wrote the manuscript.

## Conflicts of Interest

The authors declare no conflicts of interest.

## Supporting information


**Appendix 1:** A sample search strategy for CINAHL Plus. **Appendix 2:** The characteristics’ of included studies. **Appendix 3:** Critical appraisal results.

## Data Availability

The data that support the findings of this study are available from the corresponding authors upon reasonable request.
