# Supplementary material for: Supporting Adolescent Mothers to Make Infant Feeding Decisions: A Qualitative Evidence Synthesis
Source: Matern Child Nutr. 2025 Aug 29;21(4):e70098. doi: 10.1111/mcn.70098 (PMC12454177; doi:10.1111/mcn.70098)
Supplement: Supplementary file 1 — Appendix 1: A sample search strategy for CINAHL Plus. Appendix 2: The characteristics’ of included studies. Appendix 3: Critical appraisal results. [file MCN-21-e70098-s001.docx]

**Appendices**

Contents

[**Appendix 1: A sample search strategy for CINAHL Plus.** 2](#_Toc204118928)

[**Appendix 2: The characteristics’ of included studies.** 3](#_Toc204118929)

[**Appendix 3: Critical appraisal results** 16](#_Toc204118930)

# **Appendix 1: A sample search strategy for CINAHL Plus.**

Table 1. A sample search strategy for CINAHL Plus.

| Search |  |
| --- | --- |
|  | adolescent motherhoodOR adolescent mom ORyoung mothers ORadolescent mums ORyoung mum OR teenagemums OR teen moms OR Early adult mothers |
|  | (MM "Adolescent Mothers") |
|  | healthcare providers ORhealthcare workers ORphysician OR doctors OR (dieticians or nutritionists) OR registered dietitianOR lactation counsellorOR lactation counsellor OR health cadre |
|  | (MH "HealthPersonnel+") |
|  | (MH "Nurses+") |
|  | (MM "Dietitians") OR (MH"Health Educators+") |
|  | (MM "LactationConsultants") |
|  | (MH "Support Groups+") |
|  | (MM "Peer Group") |
|  | S1 OR S2 OR S3 OR S4OR S5 OR S6 OR S7 ORS8 OR S9 |
|  | (MH "Infant Feeding+") |
|  | infant fed* OR infantfeed* |
|  | breast feed* OR Breastfed* OR Breastfed |
|  | partial breastfeeding OR prelacteal feeding OR predominant feeding |
|  | (MM "Bottle Feeding") OR formula feeding ORformula fed OR formulamilk OR mixed feeding |
|  | (MM "Bottle Feeding") OR formula feeding ORformula fed OR formulamilk OR mixed feeding |
|  | (MH "Milk, Human+") |
|  | (MM "Lactation") |
|  | (MH "Breast Feeding+") |
|  | S11 OR S12 OR S13 ORS14 OR S15 OR S16 ORS17 OR S18 OR S19 |
|  | S10 AND S20 |
|  | (MH "Decision Making+") OR (MM "Decision Making, Shared") |
|  | (MH "Decision Making+") OR (MM "Decision Making, Shared") |
|  | (MM "Decision-MakingSupport (Iowa NIC)") |
|  | (MM "Decision Making,Ethical") |
|  | support* OR help ORadvice OR care OR aidOR assistance ORguidance OR Information |
|  | (MH "Counseling+") OR lactation consultation OR lactation counselling OR directive counseling |
|  | S22 OR S23 OR S24 ORS25 OR S26 OR S27 |
|  | S21 AND S28 |
|  | (MH "Qualitative Studies+") |
|  | Observation |
|  | (MH "Interviews+") |
|  | (MM "Focus Groups") |
|  | (MH "Perception+") |
|  | experiences OR (views or opinions or perceptions or beliefs) OR reflection OR feelings |
|  | (MH "Life Experiences+") |
|  | S30 OR S31 OR S32 ORS33 OR S34 OR S35 ORS36 |
|  | S29 AND S37 |

# **Appendix 2: The characteristics’ of included studies.**

Table 2. The characteristics’ of included studies.

| **Author (s) and country** | **Aim** | **Sample and setting** | **Design** | **Data Collection Methods/Instrument** | **Outcomes** |
| --- | --- | --- | --- | --- | --- |
| Acheampong et al., (2020); Ghana | To understand factors that facilitate the teenage mothers’ decision to consider exclusive breastfeeding in such a social context. | Thirty adolescent mothers aged 13 to 19 years. | Qualitative, exploratory, descriptive, and contextual design. | Focus Group Discussion. | - Positive beliefs about the benefits of breastfeeding influenced the decision to breastfeed. Participants also noted that the vicarious experiences of mothers provided positive motivation. - Participants' partners and close family members supported the decision through approval and financial assistance. - The opinions of health professionals served as motivation to breastfeed. Participants felt that midwives fully supported them and were pleased to educate mothers multiple times. |
| Amekpor et al., (2025); Ghana | This study explores the psychosocial factors affecting exclusive breastfeeding (EBF) among first-time mothers in Ghana, highlighting the challenges faced by first-time mothers in maintaining EBF for the recommended 6 months. | Twelve first-time mothers aged 19 to 35 years who were currently breastfeeding and attending post-natal clinics at Salaga Municipal Hospital, Ghana. | A qualitative exploratory design. | Semi-structured interviews. | - Adolescent mothers who are able to endure the challenges are more likely to continue breastfeeding. |
| Arthur et al., (2007); United Kingdom | - To explore teenage mothers’ experiences (accessibility and acceptability) of maternity services in the county. - To identify whether maternity services in the county meet the standards set by the Children’s and Maternity NSF. | Eight participants of teenage parents. | Phenomenology qualitative research. | Semi-structures/ conversational style interviews. | - Lack of support and preparation for breastfeeding challenges may lead to cessation, even for those who initially intended to breastfeed. |
| Astuti et al., (2021); Indonesia | To explore the experiences of breastfeeding practices among Indonesian young mothers. | Eighteen young mothers aged 15 to 19 years. | A qualitative exploratory study. | One-to-one in-depth interviews. | - Employment factors contributed to mothers ceasing breastfeeding. - The maternal grandmother was a more influential support figure than the mother’s spouse; however, in some cases, she suggested introducing additional food or milk, indicating a lack of autonomy. Mothers hoped that breastfeeding education could also be provided to their spouses and close family members for better support. - Participants mentioned receiving support from healthcare professionals and health cadres; however, mothers felt overwhelmed when they were not available. |
| Benson, (1996); Australia | To explore mothers experience of breastfeeding and motherhood. | Seventy-four mothers aged 13 to 18 years. | Qualitative study. | Interviews. | - Public breastfeeding embarrassment is one of the challenges faced by breastfeeding mothers. - The support received by mothers can vary. Some nurses are supportive, while others may pressure mothers to continue breastfeeding regardless of their condition. - Establishing breastfeeding as a norm within the family can enhance the continuity of breastfeeding. |
| Bentley et al., (1999); United States | To understand how the infant feeding decision are made and why solids are introduced. | Nineteen participants, aged 13 to 20 years, African American, first-time mothers, and eligible for WIC services. | A qualitative ethnography studies. | Semi-structured interview. | - The grandmother was the decision-maker, even if her will was contrary to the doctor’s advice. - A baby’s physical appearance can play an important role in the infant feeding practices of the mother or grandmother. |
| Bernie (2014); United Kingdom | To explore health, social, and voluntary care professionals’ perceptions of young mothers’ attitudes to breastfeeding and the role of maternal grandmothers. | A family nurse, two specialist teenage midwives, two health visitors, two voluntary group staff, and two Children’s Centre staff, all experienced in assisting mothers aged ≤ 20 years. | Qualitative study. | Semi structured interview. | - Breastfeeding decision-making challenges: social norms/approval, the breastfeeding challenges outweigh the benefits, and lack of support from HCP. - HCP tried to make AM feel less pressured. - Young mothers felt more comfortable with HCPs whom they know well. |
| Bettison (2014); United Kingdom | To explore the perceptions of health visitors who were supporting a teenage mother to breastfeed her baby, using semi-structured interviews. | Thirteen health visitors who are previously take part in the UNICEF BFI training. | Qualitative study. | Semi structured interview and FGD. | - Support and encouragement from the health visitor are vital; however, teenage mothers' past experiences, self-confidence, and support systems also influence the decision. |
| Breevort et al., (2021); Sierra Lone | To gain understanding into and explore both matters to develop recommendations for effective strategies to promote breastfeeding practice in Pujehun District, Southern Sierra Leone. | One hundred and nineteen-four mothers aged 16 to 48 years. | Mix methods. | Questionnaire, semi structured interviews, and FGD. | - Exclusive breastfeeding enablers were being informed about the benefits of breastfeeding during pregnancy, receiving support from nurses and husbands, and awareness of how friends and family fed their babies. - The primary barriers were a lack of encouragement from husbands, as well as mothers feeling that their infants' stools were abnormal or that they were not producing enough breast milk. |
| Concha & Jovchelovitch (2021); Columbia | To helps build the evidence-base for the transferability of a family-system approach to Global south regions by using sociocultural and community psychology concepts to fortify the rationale for including grandmothers in maternal and child nutrition programmes. | - Thirty-five mothers aged 16 to 29 years at prenatal and 21 mothers at post-natal. - Fifteen grandmothers at prenatal and 12 at postnatal. - Eleven peer community mother, traditional birth attendants, and community psychologists. | Qualitative longitudinal design. | Interviews. | - Grandmothers played a significant role as decision-makers in breastfeeding and infant feeding practices. |
| Condon et al. (2013) | To explore teenagers’ experiences of the breastfeeding promotion and support delivered by health professionals. | Twenty-nine pregnant and teenage mothers aged less than 18 years old. | Qualitative study. | Semi-structured interviews and FGD. | - The intention to breastfeed was influenced by several factors: health benefits, breastfeeding feasibility, spouse suggestions, embarrassment about breastfeeding in public, conflicts between personal activities and the baby’s feeding needs, and healthcare professionals' advice. - Mothers who had stopped breastfeeding often expressed regret about not having breastfed for longer. - There was a need for information to support decision-making. - Support was reported to be provided for a maximum of only two weeks. |
| Cooper et al. (2019); Tanzania | To identify barriers and facilitating factors for optimal nutrition and PPFP practices in Mara and Kagera, Tanzania. | Twenty-four mothers aged 15 to 32 years, 12 grandmothers, 6 health providers, 12 traditional birth attendants (BTA). | Qualitative study. | FGD and in-depth interviews. | - Decisions about infant feeding were made by the husband, mother-in-law, and father-in-law. Some of these decisions led to the introduction of prelacteal foods. |
| Debnath et al. (2021); Debnath | - To estimate the proportion of newborns who initiated early breastfeeding after birth. - To estimate the proportion of infants exclusively breastfed up to 42 days postpartum period. - Secondary objective: To understand the deterministic factors and barriers associated with early interruption of exclusive breastfeeding. | Three hundred and nineteen mother-newborn dyads, mean age 22.6 years. | Mix method. | Using semi structured questionnaire with open-ended questions. | - The adolescent mothers followed the cultural ritual or family member advice (e.g. give honey to the baby), indication a lack of autonomy. |
| Duong et al. (2005); Vietnam | To explore the determinants of breastfeeding practices within the first 6 months post-partum among women residing in rural Vietnam. | Four hundred and sixty-three respondents. | Longitudinal qualitative study. | FGD and interviews. | - One reason for breastfeeding cessation was the mother's perception of insufficient milk supply. |
| Dykes et al. (2003); United Kingdom | To explore the experiences and support needs of adolescent mothers who commencing breastfeeding | - Focus Group Discussion (FGD): Seven mothers aged 16 to 19 years. - Interview: thirteen adolescent mothers aged 14 to 19 years. | Multimethod qualitative study. | FGD and interviews | - Adolescent mothers (AMs) felt that older individuals and healthcare professionals were watching and judging them. For example, healthcare professionals stereotyped them as likely to bottle-feed or pressured the mothers to breastfeed. - There was a concern that feeding a baby in public could be embarrassing. - A lack of confidence in coping with breastfeeding challenges emerged when AM experienced feelings of milk insufficiency, tiredness, discomfort, and pain. - AM expressed a desire to involve their partners and reduce the feeling of being tied down. |
| Dyson et al. (2010) | - To examine the psychosocial factors influencing infant feeding intention among pregnant women expecting theory first baby. - To provide contextual insight into the interpretation and meaning of those factors. | Seventy-one pregnant teenagers aged 16 to 19 years. | Mixed method. | A quantitative questionnaire based on theory of planned behaviour and FGD. | - Formula feeding is perceived as acceptable based on moral norms, while breastfeeding mothers are often viewed as lazy for not making milk at night. - AM felt watched by the health visitor and did not feel comfortable around them. - The concept of "breastfeeding in public" is a concern for participants, highlighting the need for high levels of self-confidence to breastfeed in public. |
| Erfina et al. (2019); Indonesia | To explore adolescent mother’s postnatal inpatient experiences and healthcare needs as they moved towards their maternal roles. | Eleven adolescent mothers. | Descriptive qualitative study. | In-depth interview. | - The adolescent mothers expressed that breast milk insufficiency, baby rejection, and giving formula milk led to feelings of sorrow, guilt, and stress. - The non-rooming-in policy led to a lost opportunity for adolescent mothers to breastfeed. - Healthcare primarily focused on contraceptive counselling and physical examinations, lacking breastfeeding information and support. |
| Hannon et al. (2000); United States | To explore minority teen mothers’ perceptions of breastfeeding and the influences on infant feeding choices. | Thirty-five primiparous adolescent mothers aged 12 to 19 years. | Ethnography qualitative research. | Semi-structured ethnographic interview. | - Key factors influencing the decision to breastfeed are the benefits of breastfeeding and mother-baby bonding. - Challenges include discomfort, public exposure, difficulty with positioning and latch-on, fatigue, medical complications, and myths about breastfeeding. - Influential figures for adolescent mothers are their mothers, healthcare professionals, friends, relatives, teachers, and the baby's father. - There is a lack of support from healthcare providers in making prenatal feeding decisions. |
| Harner & McCarter-Spaulding (2004) | To explore the impact of paternal age on a teenage mother’s decision regarding infant feeding method during the postpartum hospital stay. | Eighty-six postpartum teenage mothers participated in quantitative data collection, and 34 of them participated in qualitative data collection. | A non-experimental, comparative, descriptive design. | A semi structured confidential and private interview using questionnaire. | - Husbands played a significant role in influencing mothers' infant feeding decisions, with some actively encouraging them to breastfeed. |
| Hunter (2012); United Kingdom | To explore perceptions of obstacles faced by, and support available to, teenage mothers initiating breastfeeding. | Eighty-three healthcare professionals involved in the care of pregnant and parenting teenagers in the UK. | Descriptive study. | e-questionnaire with open ended questions. | - The reasons for formula feeding included a lack of motivation, patience, and determination. - There was a discrepancy between the intentions of healthcare professionals (HCPs) to assist adolescent mothers and the adolescent mothers' perceptions of the support they received. |
| Hunter & Magill-Cuerden (2014); United Kingdom | We aimed to explore how the inpatient experiences of a group of young women who gave birth as teenagers influenced their feeding decisions and experiences,  and ascertain their ideals for breastfeeding support. | Fifteen mothers aged 16 to 20 in a young parents’ group. | A constructivist qualitative approach. | FGD. | - The first breastfeeding was generally initiated by midwives. - Healthcare professionals assumed that adolescent mothers preferred bottle-feeding and did not provide any feeding support, highlighting a communication gap between the mothers and healthcare providers. |
| Hunter et al. (2015); United Kingdom | To explore the ways in which a small group of UK adolescent mothers conceptualise their decisions to breastfeed and experience breastfeeding in their communities. | Fifteen mothers aged 16 to 20 in a young parents’ group. | A constructivist qualitative approach. | FGD. | - The decision to breastfeed was influenced by adolescent mother’s desire to validate her mothering credentials. - There was a taboo against public breastfeeding, even in front of partners. - Feelings of isolation were worsened by the perception that no support was available. |
| Hunter-Adams et al. (2022); Africa | To explore the implementation of seemingly contradictory health policies that promote exclusive breastfeeding and education policies that promote the completion of schooling for young women post-partum, and their recommendations. | Twenty-four stakeholders including nursing staff. | Qualitative study. | Guided in-depth interview. | - Adolescent mothers faced breastfeeding challenges and may alter their decisions based on advice from close family members. - An alternative approach to support is to connect mothers with community-based services or local support groups. |
| Jama et al. (2017); South Africa | To prospectively explore enablers or barriers to success among mothers who planned to exclusively breastfeed their infants for the first six months of life, in KwaZulu-Natal, South Africa. | Twenty-two mothers aged 15 to 34 years. | Qualitative longitudinal study. | Quantitative questionnaire (for baseline data) and guided in-depth interview. | - The grandmother has a significant influence on the mother's decisions. - Returning to school was one of the reasons for the cessation of breastfeeding. |
| Jama et al. (2018); South Africa | To explore autonomy and infant feeding decision-making among teenage mothers in a rural and urban setting in KwaZulu-Natal, South Africa. | Thirty participants from urban and rural areas, 10 of whom were adolescents. | A qualitative longitudinal study. | In depth interview each month for six months, starting two weeks after delivery. | - The intention to exclusively breastfeed (EBF) was based on their breastfeeding knowledge and a lack of money to buy formula. - The feeding decisions were made in the household by elders, with the teenage mothers having little or no autonomy. - Returning to school was one of the reasons for breastfeeding cessation; nevertheless, some of them were able to express breast milk at home and attend school. |
| Kocturk (1987) | To identify the underlying reasons for early complementation in areas with low to middle socioeconomics characterization. | - Quantitative: 269 mother-infant pairs. - Qualitative: Three mothers, one of whom was 19 years old. | Mix methods. | Questionnaire and case histories. | - The adolescent mother’s mother, the grandmother, assisted with the baby care and introduced prelacteal foods to the infant. This situation worsened when the doctor also suggested formula while the baby was suffering from diarrhoea. |
| Leeming et al. (2015); United Kingdom | To understand the first-time breastfeeding mothers’ experiences within the social context of feeding. | Twenty-two first-time breastfeeding mothers aged ≥16 years, including two mothers under the age of 20. | Qualitative study. | Audio diary recording and interviews. | - Adolescent mothers tended to be passive, often stopping breastfeeding when advised to do so. This led to feelings of guilt when they decide to switch to formula. |
| Locklin (1995); United States | To describe the experience of low-income women who were supported by peer counsellors/ breastfeeding advocates. | Seventeen Africa American mothers aged 18 to 37 years. | Grounded Theory qualitative study. | Observations and interviews. | - There is a possibility for mothers to change their intention from not breastfeeding to breastfeeding with support from healthcare professionals (HCPs). |
| Mazza et al. (2015); Brazil | To identify the social representations of adolescent nursing mothers on breastfeeding. | Nineteen adolescent mothers aged 10 to 19 years at the time of childbirth. | Exploratory qualitative study. | Semi-structured interview. | - Adolescent mothers seek advice from experienced individuals, such as their mothers, who serve as influential figures and provide vicarious experiences. - Healthcare professionals perceived that adolescent mothers did not want to breastfeed. - There were differing opinions among professionals regarding the management of breastfeeding. |
| Merino et al. (2013); Brazil | To understand the main situations faced by adolescents and the coping strategies they employ after the baby is born. | Seventy-eight mothers aged 14 to17 years, included in the surveillance program of the baby at risk. | Descriptive qualitative study. | Semi-structured interview. | - The mother switched the infant feeding method due to the challenges she faced. - The mother's close family provided support but also suggested additional culturally common foods and drinks, which adolescent mothers felt obligated to follow. - There was a lack of citations regarding the role of health professionals. |
| Monteiro et al. (2014); Brazil | To characterise breastfeeding practices among Brazilian adolescents and identify their breastfeeding needs. | Ten adolescent mothers (aged <19 years) in a primary care unit. | Mix-methods. | - Quantitative: secondary data analysis. - Qualitative: interview. | - The intention to breastfeed is influenced by the perceived benefits of breastfeeding for the infant’s needs. - Difficulties with breastfeeding often lead to an early introduction of solid foods, which can boost mothers' confidence regarding their infant's health. - Sources of help and support while breastfeeding come from both healthcare professionals (HCPs) and the grandmothers. |
| Moran et al. (2006); United Kingdom | To investigate the similarities and differences in the approaches of midwives and qualified breastfeeding supporters (the Breastfeeding Network (BfN)) in supporting breastfeeding adolescent mothers. | Twelve midwives, twelve BfN supporters, and seven mothers aged 16 to19 years. | Qualitative study using vignette. | FGD and vignettes. | Both midwives and BfN supporters also emphasised a caring atmosphere; that they created a comfortable environment and would support the mother with whatever decision she made, avoiding judgment and endorsing her decision. |
| Morrison et al. (2008); United States | To examine the factors that influences young mothers’ infant-feeding practices in Hillo. | Thirty-three mothers aged 16 to 27 years. | Qualitative study. | FGD and in-depth interviews. | - Influential persons for the teenage mother include her mother, grandmother, and partner. - Public embarrassment surrounding breastfeeding impacted the infant feeding process. |
| Nelson (2009); United States | To investigate the attitudes, beliefs, and concerns of pregnant and postpartum adolescents regarding breastfeeding. | Sixteen participants  Enrolled in a young-parents outreach program. | Exploratory qualitative design. | FGD. | - There was consensus that the opinions of others should not influence a mother’s choice to breastfeed. - Breastfeeding challenges, such as pain, often led mothers to stop breastfeeding. |
| Nelson & Sethi (2005); Canada | To discover the phenomenon of breastfeeding as experienced by teenage mothers. | Eight Canadian teenage mothers aged 15 to 19 years. | Grounded qualitative approach. | Informal interview and demographic questionnaire. | - The breastfeeding commitment journey is a continuous process that begins with the decision to breastfeed during pregnancy, followed by learning, adjusting, and eventually ending breastfeeding. - Mothers often choose to breastfeed because they believe it is cheaper than formula. - Participants emphasised the importance of breastfeeding support. |
| Nesbitt et al. (2012); Canada | To examine the facilitating influences and barriers to initiating, and continuing breastfeeding, as perceived by  adolescent mothers in Durham Region, Ontario, Canada. | Sixteen adolescents aged 17 to 19. | Qualitative study. | Individual, semi-structured, face to face interviews. | - The decision to initiate breastfeeding occurred during the prenatal period, influenced by partners and family but ultimately made independently by the mother. - Factors affecting the continuation of breastfeeding included its impact on relationships, social support availability, physical demands, and maternal comfort. - Participants expressed a lack of knowledge about breastfeeding norms and felt no need for professional support, though some were aware of community-based services. |
| Nuampa et al. (2018); Thailand | To explore Thai adolescent mothers’ experiences related to breastfeeding for the first six months. | Twenty adolescent mothers aged 15 to 19 years. | A descriptive qualitative study. | semi-structured, face to face interviews. | - Reasons for breastfeeding included improving infant health, benefits for mothers, enhancing maternal perception, cost savings, and family motivation to breastfeed. - Challenges faced included the embarrassment of breastfeeding in public and the difficulties of returning to work or school. |
| Oliveira et al. (2016); Brazil | To know the perception of adolescent mothers of the causes that influence the interruption of exclusive breastfeeding. | Fourteen first-time young mothers, aged 10 to 19 years, did not practice exclusive breastfeeding (EBF). | Descriptive study. | Semi-structured interview. | Reasons for the interruption of exclusive breastfeeding (EBF) include persistent crying, availability of support, challenges with latch and positioning, the need to work, family beliefs and cultural myths, and lack of professional support. |
| Pentecost & Grassley (2014); United States | To explore the needs of adolescent for social support from nurses when initiating breastfeeding. | Ninety adolescent mothers aged 15 to 20 years. | A secondary qualitative content analysis. | The data were obtained as a part of psychometric study to evaluate a scale designed to measure adolescent’s perceptions of nurses’ breastfeeding support in the immediate postpartum. | The participants mentioned the support from HCPs on how to breastfeeding, answering their questions, and provide encouragement. |
| Raisler (2000); United States | To explore the experiences of  low-income nursing mothers within and beyond the  health care system. | Forty-two women aged 16 to 39 years participated in the WIC program. | Qualitative Research. | FGD. | - AM felt her breastfeeding wishes were discounted due to her age. - She faced overwhelming health information and emphasised the need for a more personal approach. |
| Rothstein et al. (2020); Peru | - To characterize patterns of mixed feeding during infants’ first two months of life. - To evaluate how interactions among health providers, formula company representatives, and mothers shape those practices. | Two hundreds and fourteen mothers, mean age 27.9 years. | Cohort mix methods. | Infant-feeding surveillance, semi structured questionnaire and in-depth interviews. | - The study reported a lack of support from healthcare professionals (HCPs). |
| Severinsen et al., (2024)**;** New Zealand | to explore the experiences of young mothers who exclusively breastfed for six months or longer, focusing on how they encountered stigma and expressed resistance via social media. | Forty-four young mothers who were breastfeeding for six months and longer, aged 18 to 24 years, living in Aotearoa New Zealand. | A qualitative study. | In-depth, semi-structured telephone interviews. | - Adolescent mothers encountered challenges due to the influence of family members on their feeding decisions and often felt unable to disregard these suggestions, even when they conflicted with their own beliefs. - One mother reported receiving misleading advice from healthcare professionals, who claimed that breastfeeding did not provide sufficient nutrition. |
| Smith et al. (2012); United States | To understand the factors that contribute to the breastfeeding decision and practices of teen mothers. | Seventeen pregnant teens, aged 14 to 17, enrolled a seven-week child-birth education program. | A prospective qualitative study. | A semi structured in-person baseline interview. | - The perception that "breast is best" was a key rationale behind the teens' intention to breastfeed. - Breastfeeding challenges faced by adolescent mothers included returning to school, inappropriate suggestions from healthcare professionals, and social embarrassment. - There was a lack of knowledge and skills to overcome these challenges, even though they received breastfeeding education as part of their childbirth classes. |
| Spear (2006); United States | To examine the breastfeeding experiences and related behaviours of adolescent mothers after discharge from the hospital. | Fifty-three adolescent mothers aged 14 to 19 years. | A cross-sectional, descriptive study. | Telephone survey with close and open-ended questions. | - Challenges of breastfeeding, such as inadequate milk supply, sore nipples, and responsibilities related to work or school, often lead to weaning. - Participants had a positive view of the support from healthcare professionals (HCPs); however, they suggested more discussions and clearer information about the advantages of breast milk. - Follow-up breastfeeding support after hospital discharge was deemed essential. |
| Spindola et al. (2014); Brazil | To identify the factors that influence on primiparous adolescent mothers’ breastfeeding and to comprehend the meaning of it to this woman. | Fourteen primiparous adolescent mothers aged 10 to 19 years who attended the appointments routinely. | Descriptive qualitative study. | Open, individual interview. | - Health care professionals (HCPs) should provide guidance while respecting differing views. Therefore, the mother was not compelled to breastfeed against her will. - Important factors in the decision included family support and vicarious experiences. - There was a varying level of support from HCPs, and a good relationship between the mother and HCP was essential. |
| Tomeleri & Marcon (2009); Brazil | To identify and describe popular practices of teenage mothers caring for their children during the first 6 month after birth. | Six adolescent mothers aged 15 to18 years. | Qualitative research. | Interviews. | - Mothers were mainly influenced by their own mothers and close relatives. - They received most breastfeeding support from family, while there were indications that healthcare professionals provided misleading information. |
| Tucker et al. (2011); United States | To investigate breastfeeding practices, barriers, and facilitators among adolescent mothers aged 17 and younger. | - Quantitative analysis: 389 mothers. - Qualitative analysis: twenty-two adolescent mothers aged 13 to 17 years. | Mix methods. | - Quantitative: secondary data analysis. - Qualitative: Semi-structured interview. | - The reasons mothers changed their decision included negative influences from friends and relatives, physical discomfort, insufficient milk supply, persistent crying from the baby, and returning to school. - There was a lack of support from healthcare professionals (HCPs) after hospital discharge. |
| Wambach & Cohen (2009); United States | To examine the breastfeeding experiences of urban adolescent mothers. | Twenty-three teens aged 14 to 18 years. | A qualitative descriptive study. | FGD and semi-structured interview. | - Some participants made their decision to breastfeed prenatally, involving a process of reflection, information gathering through reading, videos, and classes, and discussions with influential figures like family, friends, and healthcare providers. - Returning to school was a reason for weaning. - A few reported negative breastfeeding supports, feeling ignored, receiving contradictory information, and being advised to give infants formula. |
| Wambach & Koehn (2004); United States | To report a pilot study of influencing factors in  disadvantaged urban pregnant adolescents’ decision-making about infant-feeding  choices. | Fourteen Pregnant adolescent aged 14 to 18 years. | Qualitative research. | FGD. | - The decision on how to feed their babies was ultimately the mothers' own choice, influenced by advisors like their mothers, sisters, and birthing teachers. - The perceived complexity of breastfeeding created uncertainty about exclusive breastfeeding, affecting decisions to combine breast- and bottle-feeding. - Social embarrassment emerged as an issue for teenage mothers, and none reported direct influence from healthcare professionals. |
| Wambach et al. (2016); c | To identify perceived benefits and barriers to exclusive breastfeeding and level of acculturation among Mexican American women living in a midwestern city. | Twenty-one Mexican American women aged 16 to 39. | A descriptive qualitative study. | Semi-structured interview. | - A mother with greater support for breastfeeding is likely to breastfeed for a longer period. - Adolescent mothers tend to follow the advice of elders or healthcare professionals. |
| (Yas et al., 2024)**;** United States | To investigate the perceived standard needs of adolescent mothers during breastfeeding from the viewpoint of healthcare providers. | Fourteen HCPs worked in Mashhad and Urmia Province, Iran. | A qualitative content analysis. | In person, semi-structured interviews. | - Healthcare providers in this study observed that adolescent mothers face breastfeeding challenges due to cultural misconceptions (e.g., colostrum being harmful, small breasts unable to produce milk) and a lack of support. - Many receive little to no breastfeeding education during pregnancy. Providers emphasised the need for tailored education through schools, antenatal care, and media. Emotional support and confidence-building are also vital, as many young mothers lack self-efficacy and may discontinue breastfeeding without adequate encouragement. |

# **Appendix 3: Critical appraisal results**

Table 3. The critical appraisal results

| **No.** | **Author** | **1** | **2** | **3** | **4** | **5** | **6** | **7** | **8** | **9** | **10** |
| --- | --- | --- | --- | --- | --- | --- | --- | --- | --- | --- | --- |
|  |  | **Was there a clear statement of the aims of the research** | **Is a qualitative methodology appropriate** | **Was the research design appropriate to address the aims of the research?** | **Was the recruitment strategy appropriate to the aim of the research?** | **Was the data collected in a way that addressed the research issue?** | **Has the relationship between researcher and participants been adequately considered?** | **Have ethical issues been taken into consideration?** | **Was the data analysis sufficiently rigorous?** | **Is there a clear statement of findings?** | **How valuable is the research?** |
|  | Acheampong et al., (2020) | Yes | Yes | Can’t tell | Can’t tell | yes | No | yes | yes | No | * |
|  | Amekpor et al. (2023) | Yes | Yes | Yes | Yes | Yes | Yes | Yes | No | Yes | *** |
|  | Arthur et al., (2007) | Yes | Yes | Yes | Can’t tell | Yes | No | Yes | Yes | Yes | * |
|  | Astuti et al., (2021) | Yes | Yes | Can’t tell | Yes | Yes | Yes | Yes | Yes | Yes | *** |
|  | Benson, (1996) | Yes | Yes | Yes | Can’t tell | Can’t tell | Can’t tell | Can’t tell | Can’t tell | Can’t tell | ** |
|  | Bentley et al., (1999) | Yes | Yes | Yes | Can’t tell | Yes | No | No | Can’t tell | Yes | ** |
|  | Bernie (2014) | Yes | Yes | Yes | Can’t tell | Can’t tell | No | Can’t tell | Can’t tell | Yes | *** |
|  | Bettison (2014) | Yes | Yes | Yes | Yes | Can’t tell | No | Yes | Yes | Can’t tell | ** |
|  | Breevort et al., (2021) | Yes | Yes | Can’t tell | Yes | Yes | No | Yes | Yes | Yes | *** |
|  | Concha & Jovchelovitch (2021) | Yes | Yes | Can’t tell | Can’t tell | Can’t tell | Yes | Yes | Yes | Yes | * |
|  | Condon et al. (2013) | Yes | Yes | Can’t tell | Can’t tell | Can’t tell | No | Yes | Can’t tell | Can’t tell | *** |
|  | Cooper et al. (2019) | Yes | Yes | Can’t tell | Can’t tell | Yes | Yes | Yes | Yes | Yes | ** |
|  | Debnath et al. (2021) | Yes | Yes | Can’t tell | Yes | Yes | No | Yes | Can’t tell | Yes | * |
|  | Duong et al. (2005) | Yes | Yes | Can’t tell | No | Can’t tell | No | Yes | Can’t tell | Yes | ** |
|  | Dykes et al. (2003) | Yes | Yes | Can’t tell | Yes | Yes | Yes | Yes | Yes | Yes | *** |
|  | Dyson et al. (2010) | Yes | Yes | Can’t tell | Can’t tell | Yes | No | Can’t tell | Yes | Yes | ** |
|  | Erfina et al. (2019) | Yes | Yes | Yes | Yes | Yes | No | Yes | Yes | Yes | *** |
|  | Hannon et al. (2000) | Yes | Yes | Yes | Yes | Yes | Yes | Yes | Can’t tell | Can’t tell | *** |
|  | Harner & McCarter-Spaulding (2004) | Yes | Yes | Can’t tell | Can’t tell | yes | No | yes | Can’t tell | No | ** |
|  | Hunter (2012) | Yes | Yes | Yes | Yes | Yes | Yes | Yes | Yes | Yes | *** |
|  | Hunter & Magill-Cuerden (2014) | No | Yes | Can’t tell | Can’t tell | Yes | No | Can’t tell | Can’t tell | Yes | * |
|  | Hunter et al. (2015) | Yes | Yes | Yes | Yes | Yes | No | Yes | Can’t tell | Yes | ** |
|  | Hunter-Adams et al. (2022) | Yes | Yes | Yes | Yes | Yes | No | Yes | Yes | Can’t tell | ** |
|  | Jama et al. (2017) | Yes | Yes | Yes | Yes | Yes | No | Yes | Yes | Yes | ** |
|  | Jama et al. (2018) | Yes | Yes | Yes | Yes | Yes | Yes | Yes | Yes | Yes | ** |
|  | Kocturk (1987) | Yes | Yes | Can’t tell | Can’t tell | Can’t tell | No | No | No | Can’t tell | * |
|  | Leeming et al. (2015) | Yes | Yes | Can’t tell | Can’t tell | Yes | No | Can’t tell | Yes | Yes | * |
|  | Locklin (1995) | Yes | Yes | Yes | Yes | Yes | No | No | yes | yes | * |
|  | Mazza et al. (2015) | Yes | Yes | Can’t tell | Yes | Can’t tell | No | Yes | Can’t tell | No | * |
|  | Merino et al. (2013) | Yes | Yes | Can’t tell | Yes | Can’t tell | No | Yes | Can’t tell | Can’t tell | * |
|  | Monteiro et al. (2014) | Yes | Yes | Can’t tell | Yes | Yes | No | Yes | Can’t tell | Can’t tell | ** |
|  | Moran et al. (2006) | Yes | Yes | Yes | Yes | Yes | No | Yes | Yes | Yes | *** |
|  | Morrison et al. (2008) | Yes | Yes | Yes | Yes | Yes | No | Can’t tell | Yes | Yes | ** |
|  | Nelson (2009) | Yes | Yes | Can’t tell | Can’t tell | Can’t tell | No | Yes | Can’t tell | Yes | *** |
|  | Nelson & Sethi (2005) | Yes | Yes | Yes | Yes | Yes | No | Yes | Yes | Yes | *** |
|  | Nesbitt et al. (2012) | Yes | Yes | Yes | Yes | Yes | Yes | Yes | Yes | Yes | *** |
|  | Nuampa et al. (2018) | Yes | Yes | Yes | Yes | Yes | No | Yes | Yes | Yes | ** |
|  | Oliveira et al. (2016) | Yes | Yes | Yes | Can’t tell | Can’t tell | Can’t tell | Yes | Can’t tell | Can’t tell | * |
|  | Pentecost & Grassley (2014) | Yes | Yes | Can’t tell | Can’t tell | Can’t tell | No | Can’t tell | Yes | Yes | *** |
|  | Raisler (2000) | Yes | Yes | Yes | Can’t tell | Can’t tell | No | Can’t tell | Can’t tell | Can’t tell | * |
|  | Rothstein et al. (2020) | Yes | Yes | Yes | Yes | Yes | No | Yes | Yes | Yes | *** |
|  | Severinsen et al. (2024) | Yes | Yes | Yes | Yes | Yes | Can’t tell | Yes | Yes | Yes | *** |
|  | Smith et al. (2012) | Yes | Yes | Can’t tell | Yes | Can’t tell | No | Yes | Yes | Yes | ** |
|  | Spear (2006) | Yes | Yes | Yes | Can’t tell | Yes | No | Yes | Can’t tell | Yes | *** |
|  | Spindola et al. (2014) | Yes | Yes | Yes | Can’t tell | Yes | No | Yes | Can’t tell | Can’t tell | * |
|  | Tomeleri & Marcon (2009) | Yes | Yes | Can’t tell | Can’t tell | Can’t tell | No | Yes | Can’t tell | Yes | * |
|  | Tucker et al. (2011) | Yes | Yes | Can’t tell | Yes | Yes | No | Can’t tell | Yes | Yes | *** |
|  | Wambach & Cohen (2009) | Yes | Yes | Yes | Can’t tell | Yes | No | Yes | Yes | Yes | ** |
|  | Wambach & Koehn (2004) | Yes | Yes | Can’t tell | Can’t tell | Can’t tell | Can’t tell | No | Yes | Yes | *** |
|  | Wambach et al. (2016) | Yes | Yes | Can’t tell | Yes | Yes | No | Can’t tell | Yes | Yes | ** |
|  | Yas et al. (2024) | Yes | Yes | Can’t tell | Can’t tell | Can’t tell | Can’t tell | Yes | Yes | Yes | * |
| Q10:  (*) If one of the criteria was addressed.  (**) If two of the criteria was addressed.  (***) If all of the criteria (3) was addressed | | | | | | | | | | | |
